# Programmable Vanishing Multifunctional Optics

**DOI:** 10.1002/advs.201801746

**Published:** 2018-12-27

**Authors:** Xiaoqing Cai, Zhitao Zhou, Tiger H. Tao

**Affiliations:** ^1^ State Key Laboratory of Transducer Technology Shanghai Institute of Microsystem and Information Technology Chinese Academy of Sciences Shanghai 200050 China; ^2^ School of Graduate Study University of Chinese Academy of Sciences Beijing 100049 China; ^3^ School of Physical Science and Technology ShanghaiTech University Shanghai 200031 China; ^4^ Center of Materials Science and Optoelectronics Engineering University of Chinese Academy of Sciences Beijing 100049 China

**Keywords:** diffractive optical elements, multichromatic, multilevel, programmable vanishing

## Abstract

Physically transient optics, a form of optics that can physically disappear with precisely controlled degradation behaviors, has widespread applications including information security, drug release, and degradable implants. Here, a set of silk‐based programmable vanishing, biologically functional, multichromatic diffractive optical elements (MC‐DOEs) is reported. Silk proteins produced by silkworms and spiders are mechanically robust, biocompatible, biodegradable, and importantly, optically transparent, which open up new opportunities for a set of fully degradable transient optical devices with no need of metallic or semiconductor components. Compared with monochromatic DOEs, MC‐DOEs carry out richer information for more practical applications such as encryption and decryption of multilevel information, quantitative sensing/monitoring of chemical/biological cascade reactions, and effective treatment of infections caused by multiple pathogens.

Recently, physically transient devices—a form of devices that can physically disappear in aqueous solutions or biofluids—have attracted extensive attention for their great potentials in biomedicine, medical implants, and information security. Various degradable polymers—including naturally extracted and synthetic ones such as proteins, polysaccharides, polycaprolactone (PCL), polyglycolic acid (PGA), and polylactic acid (PLA)—have been explored as the substrates and encapsulating materials that can dissolve in the body or the environment in a controlled fashion.^1^, ^2^, ^3^, ^4^, ^5^ However, the incompatibility of these materials with semiconductor processing for transient devices—electronic ones in particular—can limit engineering options in function, durability, and processing/manufacturing schemes.^6^, ^7^, ^8^ Particularly, the tendency of polymers to swell and crack the electronic materials when immersed in water or biofluids hinders their practical applications, where organic polymers and inorganic semiconductors/metals are integrated during the fabrication of nearly all types of active and passive electronic components, including resistors, inductors, capacitors, antennas, transistors, and diodes.^9^, ^10^, ^11^, ^12^

On the other hand, transient optical devices have advantages such as simple configuration, high speed and wide bandwidth, and robustness under complex biological conditions which are usually wet and curved. And importantly, transient optics has less power requirements—biodegradable power sources still remain as a major challenge for transient medical devices—offering opportunities for their uses in many scenarios where light needs to be precisely propagated, directed, or modulated in the free space or within the biological object, preferably at multiple wavelengths. Many biopolymers have been explored as the enabling/function material for optical devices and components in a variety of scenarios in addition to providing device support and encapsulation.^13^, ^14^, ^15^ In this context, silk proteins produced by silkworms and spiders are mechanically robust, biocompatible, biodegradable, and importantly, optically transparent, which open up new opportunities for a set of fully degradable transient optical devices with no need of metallic or semiconductor components. For example, Zhou et al. reported a set of silk‐based diffractive optical elements (DOEs) that can shape monochromatic beams into arbitrary patterns.^16^ Though initial demonstrations are appealing and promising, they still suffer from drawbacks such as limited functionalities, inability to operate at multiple wavelengths, and lack of precise control on the degradation behaviors, resulting in severe restrictions in their use in medical implants. New strategies are highly desired for physically transient optical devices consisting of functional components that can operate at multiple wavelengths and degrade in a well‐controlled fashion. This can offer substantially higher information richness and thus greater flexibilities for device design and operation.

In this paper, we report a new set of programmable vanishing multifunctional optics that is biologically functional and biodegradable with precisely controlled degradation behaviors. For a proof‐of‐principle demonstration, we fabricate a group of silk‐based biologically functional multichromatic diffractive optical elements (MC‐DOEs) which can work at multiple wavelengths either in sequence or simultaneously on demand as a customizable bioactive platform. Compared with monochromatic DOEs, MC‐DOEs carry out richer information for more practical applications such as encryption and decryption of multilevel information, quantitative sensing/monitoring of chemical/biological cascade reactions, and effective treatment of infections caused by multiple pathogens.

DOEs operate by illuminating the grating with light and characterizing the resulting diffraction pattern. Compared with conventional DOEs, which can only operate at single wavelengths, MC‐DOEs offer considerably richer information processed at multiple light channels (**Figure** **1**a). Each channel/wavelength can be programmed to operate independently or synergistically, which empowers the precise processing of multidimensional information both spatially and temporally. In this work, three channels (i.e., 445 nm for blue, 532 nm for green, and 650 nm for red, respectively) are used to evaluate the multichromatic diffraction patterns by using an RGB color model as the standard color palette with 256 color levels from 0 to 255.^17^, ^18^

**Figure 1:**
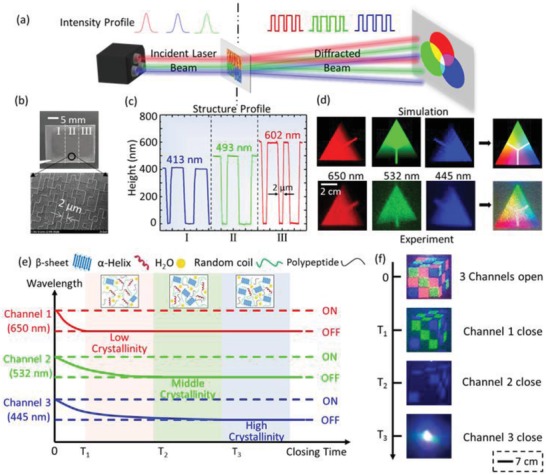
a) Schematic of the working principle of a multichromatic diffractive optical element (MC‐DOE) made by regenerated silk fibroin films. b) The fabricated silk MC‐DOE consists of three components with the operating wavelengths of 445, 532, and 650 nm, respectively. The SEM image of component II indicates the critical size (i.e., the minimum pixel size) of 2 µm. c) Characterization of three components of the silk MC‐DOE with depth levels of 413, 493, and 602 nm, respectively. d) Diffractive “color card” patterns (Top: simulated results; Bottom: experimental results) generated by a silk MC‐DOE when illuminated by three lasers with the wavelengths of 650, 532, and 445 nm, which build an RGB color model with 256 color levels. e) Diagram of the relationship between switch‐off time of the three information channels and crystallinity levels of the corresponding components of the silk MC‐DOE. As the crystallinity of the silk DOE decreases, the switch‐off time of corresponding information channel reduces. f) As a proof‐of‐principle demonstration, a programmable vanishing “magic cube” has been implemented.

The design and optimization of the diffractive microstructures in silk DOEs are carried out by a commercial software LightTrans VirtualLab, which simulates the inverted phase propagation from the diffractive plane to the structure plane by an inverse Fourier transformation.^19^, ^20^ The silk‐based MC‐DOEs are fabricated by a simple cast‐and‐peel soft lithography process, where the aqueous silk fibroin solution is cast and dried on the patterned silicon wafer (Figure S1, Supporting Information). Then, the microstructures with the critical dimension of 2 µm can be precisely duplicated. The resulting silk MC‐DOE devices have excellent biocompatibility, high transmission, robust mechanical properties, and controllable degradation^21^, ^22^ (Figure 1b). The depth levels of silk‐based components are optimized to be 413, 493, and 602 nm, corresponding to a phase shift of π at λ = 445, 532, and 650 nm with a refractive index of *n*_silk_ = 1.54,^23^ respectively (Figure 1c). The silk‐based MC‐DOE can direct beams (in R, G, B) into arbitrary patterns (i.e., color, shape, and intensity), providing a huge and editable data repository (Figure 1d and Figure S2, Supporting Information).

For actualizing programmable vanishing process, it is vital to achieve the maneuverable control of information channels to process multilevel information. By regulating the crystallinity of the silk MC‐DOE precisely (Figure S3, Supporting Information), the degradation rate and sequence of the microstructures on each component can be controlled,^24^ thereby enabling flexible design of the switch‐off time of relevant information channel. For example, degrading the silk‐based MC‐DOE with different crystallinity levels, where the component 1 (i.e., channel 1) has the lowest crystallinity (thus the fastest degradation rate), followed by channel 2 and channel 3, the switch‐off time of each channel reduces as the crystallinity of the corresponding silk DOEs decreases (Figure 1e,f).

As shown in **Figure** **2**, multilevel all‐optical information encryption/decryption, including data encryption and decryption at multiple channels, is enabled by silk‐based degradable multichromatic optics. Multiple channels in terms of different wavelengths/colors can be stored in MC‐DOE and the information of each channel can be independently programmed to conceal or reveal (Figure S4, Supporting Information). For a three‐channel configuration, the operation of the device is based on the choice of component material, each of which has agile permutation and combination to satisfy the needs of multilevel information encryption and decryption processing for potential information security applications (Figure 2a). It can also be programmed according to the requirement of the information treatment (Figure S5, Supporting Information).

**Figure 2:**
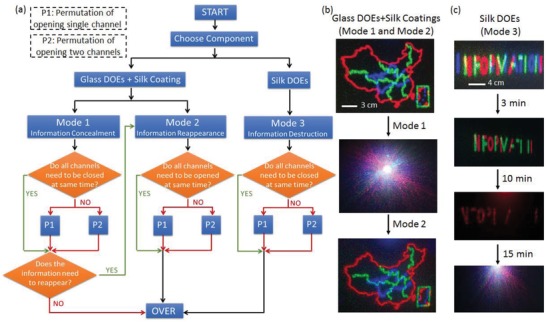
a) Glass MC‐DOE with silk coatings and all‐silk‐based MC‐DOE can be used to process the optical information in three modes, including multilevel information encryption, decryption, and destruction. Silk is optically transparent and has a refractive index close to crown glass in the visible frequencies (1.54 vs 1.52). The glass MC‐DOE with silk coatings can work in mode 1 and mode 2, which are reversible to achieve the concealment and reappearance of the multilevel information by selectively casting/removing the silk coatings (with predesigned crystallinity) onto/from the glass DOE. The silk MC‐DOE works in mode 3, in which the components with predesigned crystallinity can be selectively destroyed to realize part or entire destruction of the multilevel information. b) A map of China is stored in a glass MC‐DOE. The multilevel information of the map is concealed when the silk coatings are cast onto the glass MC‐DOE to form an “optically homogenous” medium (i.e., mode 1). By selectively removing the silk coatings from the glass MC‐DOE, the multilevel information can reappear in a predesigned order (i.e., mode 2). c) As an example of mode 3, a silk MC‐DOE storing the word “information” is customized with different crystallinity levels in its three components. When the trigger activates, the three channels are destroyed at 3, 10, and 15 min, respectively, achieving camouflage and complete destruction of the information.

Silk has a refractive index (≈1.54, in the visible regime) very close to a typical crown glass (≈1.52), which allows for multilevel information reappearance and concealment (mode 1 and mode 2) by utilizing a controllable dissolved silk protein coating.^25^ Three operation modes can be achieved, namely, information concealment (mode 1), information reappearance (mode 2), and information destruction (mode 3). As shown in Figure 2a, by applying silk protein coatings with different crystallinity levels on the surface of glass MC‐DOEs, the multilevel information stored in the component can be hidden in one or more channels. By degrading the silk protein coating sequentially, the selective reappearance of multilevel information (i.e., mode 2) can be achieved. When the multilevel information needs to be completely destroyed after reading (i.e., mode 3), a silk MC‐DOE is chosen, in which the specific region of the silk MC‐DOE is triggered to change the data in the corresponding channel to achieve camouflage or complete destruction of the information (Figure S6, Supporting Information). Note that the destruction, concealment, and reproduction of information on each level can be precisely adjusted via regulating the crystallinity levels within the silk matrix, which determines each mode's “life time,” ranging from minutes to months.^26^

The flexibility of multiple operation modes of as‐reported programmable vanishing MC‐DOEs offers a powerful way to perform multilevel information encryption and decryption. For instance, if the information needs to be read and encrypted multiple times, a glass MC‐DOE can be used to work in mode 1 and mode 2 in addition to silk coatings. As shown in Figure 2b, a map of China is stored in a glass MC‐DOE and then transmitted in three channels. By regulating the crystallinity of silk layers coated on the glass MC‐DOEs, the concealment and encryption of multilevel information can be programmed with a predesigned order. The silk coatings with different crystallinity levels are degraded by immersing them into the proteinase K solution with a concentration of 0.3 mg mL^−1^ for a suitable duration. For information that needs to be destroyed after reading, the microstructures on the surface of the silk protein MC‐DOE are sequentially destroyed. For example, the word “information” was stored in a silk MC‐DOE and three channels were destroyed at 3, 10, and 15 min, respectively (Figure 2c).

In addition to multilevel information processing, silk is renowned to be able to maintain and preserve the biochemical function of labile biological components (e.g., enzymes, antibodies, and antibiotics) within the material matrix for biochemical applications.^27^ In this work, quantitative sensing/monitoring of chemical/biological cascade reactions is enabled by the abilities to embed biochemical functions within the protein‐based material system for the direct incorporation of biological and chemical activities in as‐fabricated optical devices. We prepared a set of silk MC‐DOEs embedded with glucose oxidase (GO*x*) and horseradish peroxidase (HRP) for glucose detection. GO*x* is an oxidoreductase that can catalyze the oxidation of glucose to H_2_O_2_ and glucono‐δ‐lactone. Coupled with HRP‐catalyzed reaction, colorless transparent o‐dianisidine (ODA) is oxidized to reddish‐brown oxidized anisidine, the optical density (OD) of which can be gauged at 460 nm by a spectrophotometer.^28^, ^29^

A silk MC‐DOE consists of three components (numbered I, II, and III) that are doped with HRP, glucose, and GO*x*, respectively. It was degraded in proteinase K solution for a preset time, resulting in a HRP‐containing solution (from component I), a glucose‐containing solution (from component II), and a GO*x*‐containing solution (from component III). The concentration of glucose solution can be measured by measuring the optical density of the ODA solution reacted with the solution containing reaction products from component I, II, and III (**Figure** **3**a). The release of each biological component can be obtained by the corresponding color level value of the multichromatic diffraction pattern during the degradation of the MC‐DOE (Figures S7 and S8, Supporting Information). For example, the component II was degraded within a period of 50 min and the *R* value of the diffractive pattern is measured every 5 min, while 600 µL glucose‐containing solution is taken to react with the other two kinds of enzyme‐containing solution. Multichromatic diffraction patterns and the microstructures of the component II at different time points are shown in Figure 3b. By calibrating the mixed solution obtained at different time points by the standard concentration of glucose solution (Figures S9 and S10, Supporting Information), a one‐to‐one correspondence between *R* value and concentration of glucose solution at different time points can be obtained, providing a model for monitoring the release of glucose (Figure 3c).

**Figure 3:**
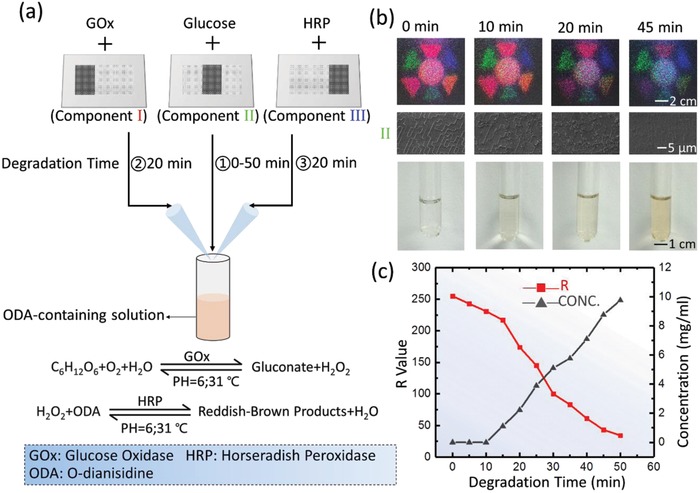
a) Silk MC‐DOEs can be used in complex cascade reaction for quantitative sensing of the concentration of final reaction product. The Silk MC‐DOE is separately doped with horseradish peroxidase (HRP), glucose, and glucose oxidase (GO*x*) in its three components (labeled as Component I, Component II, and Component III). Each component is degraded for different duration (I: 20 min, II: 0–50 min, III: 20 min) in proteinase K solution with a concentration of 0.3 mg mL^−1^. 600 µL HRP‐containing solution and 600 µL glucose‐containing solution are added to 2.5 mL o‐dianisidine (ODA)‐containing solution, warming in a 31 °C water bath for 3 min, and then adding 600 µL GO*x*‐containing solution into the mixed solution, warming in a 31 °C water bath for 2 min. b) As the silk MC‐DOE gradually degrades, the integrity of multichromatic diffraction patterns (Top part) and microstructures (Middle part, taking component II as an example) decrease, and the final reaction solution turns reddish‐brown (Bottom part). c) *R* value of the diffractive pattern of component II and concentration of glucose solution measured at different time points. The concentration of glucose solution can be derived from *R* value.

Furthermore, as shown in **Figure** **4**, in vivo monitoring of multiple drug releasing and in situ treatment of the infection caused by multiple pathogens can be enabled by embedding multiple therapeutic molecules (i.e., antibiotics) within one single optical device and operating at multiple channels for real‐time monitoring, which is difficult to achieve otherwise. The silk MC‐DOE is doped with antibiotics of gentamicin (G) and metronidazole (M) to serve as an antibacterial skin patch with the optical access to monitor the release of multiple drugs during device degradation in the reflection mode. Two kinds of bacteria (*Staphylococcus aureus* (*S. aureus*) and *Escherichia coli* (*E. coli*)) were used to infect the back of Sprague–Dawley rats (Figure S11, Supporting Information). Devices were water‐annealed for 1.5 h before being applied to the skin of Sprague–Dawley rats, and then the real‐time release of the antibiotics can be readily read out by monitoring the reflected multichromatic diffraction patterns (in red and green) while the devices are applied to the skin (Figure 4a). The silk‐DOEs were removed when the diffraction patterns disappeared (which indicated the successful release of the antibiotics) before the wounds were stitched (Figure 4a,b). Visual examination and fluorescence images of the bacteria in the animals indicated that gentamicin and metronidazole have specific therapeutic effects on *S. aureus* and *E. coli*, respectively, and the best treatment of the infection was achieved when two kinds of antibiotics worked together (Figure 4c,d).

**Figure 4:**
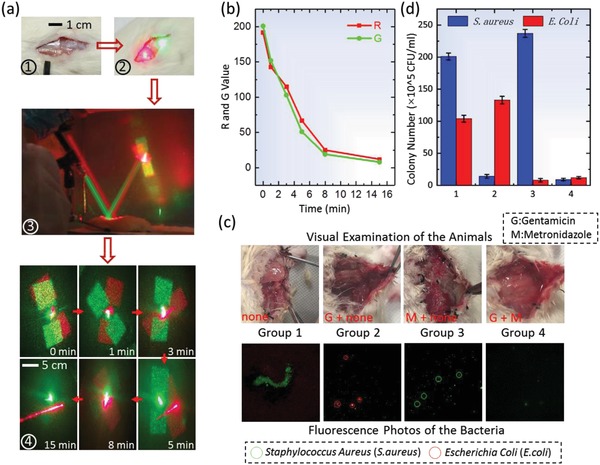
a) The silk MC‐DOE can be used as antibacterial skin patch for multiple drugs release monitoring and multiple pathogens infection treatment when working in the reflection mode. The skin of the rat is cut to create a wound that is further infected with *S. aureus* and *E. coli*. The silk MC‐DOE is then placed at the infection site on the wounded skin for 15 min (①, ②, and ③) when the diffraction patterns disappeared due to degradation (④). b) *R* and *G* values of the reflective diffraction patterns of two components of the silk MC‐DOE (doped with gentamicin and metronidazole, respectively) decrease as the silk MC‐DOE gradually degrades. c) Visual examination of the rats treated with different methods (i.e., without antibiotic, with antibiotic of gentamicin only, with antibiotic of metronidazole only, and with antibiotics of gentamicin and metronidazole) and fluorescence images of the bacteria (after 2 d) indicated effectiveness of the therapy in wound healing. d) The infected tissues of the rats were collected (after 2 d) and assessed by counting the normalized number of colony‐forming unit (CFU) in the homogenates (*n* = 4) using standard plate counting methods.

In summary, we report a new set of physically transient multichromatic optics that can direct beams at multiple wavelengths and degrade in a precisely controlled manner. Each channel/wavelength can be programmed to operate independently or synergistically. The flexibility of operation empowers the precise processing (e.g., encryption and decryption) of multidimensional information both spatially and temporally. The abilities to embed physiochemical functions within the biopolymer‐based material system enable the direct incorporation of biological activities in as‐fabricated optical devices for sensing or therapeutic purposes, which is difficult to achieve otherwise. Present sustainable development strategies, especially in the field of optical communication and optical interconnection, impose great demand for biodegradable and environmentally friendly optical materials. The usage of biopolymers as the functional material for optical devices and components such as waveguides, lens, mirrors, prisms, and diffractive elements provides a credible and sustainable alternative to current plastic, semiconductors, and inorganic substrates. We envision this technology will enable a series of biopolymers for their use across several disciplines including information security, drug delivery, implantable optical devices, and biodissolvable/degradable devices.

## Experimental Section

*Silk Solution Preparation*: The established purification protocols were used to prepare silk fibroin.^30^ Bombyx mori cocoons were boiled for 30 min in aqueous 0.02 m Na_2_CO_3_ (Sigma‐Aldrich, USA) and then rinsed for 3 × 30 min in distilled water to remove the Na_2_CO_3_ and sericin. The degummed cocoons were dried for more than 12 h and then subsequently dissolved in 9.3 m LiBr (Sigma‐Aldrich, USA) solution at 60 °C for 4 h. The solution was dialyzed for 2 d in distilled water by using Slide‐a‐Lyzer dialysis cassettes (MWCO 3500, Pierce, USA) and then centrifuged for 2 × 20 min at 18 000 rpm. The final concentration was roughly 6.9 wt%, which was determined by measuring a volume of solution and the final dried weight.

*MC‐DOE Design and Optimization*: A commercial optical simulation software LightTrans VirtualLab 5 was used for simulation to generate diffractive diffusers that could realize deterministic scattering of light into an arbitrary 2D light pattern. The desired light pattern from bitmap files was imported to get the diffractive diffusers presented in GDSII format (which could be used as the mask to define the pattern of MC‐DOE). In the simulation, the wavelength of the input beam was set as 650 nm at first, and the optical setup was chosen as paraxial far field in the simulation. The number of phase levels was set as 2 for simplification, so only a single phase mask was required. The minimum pixel size was set to 2 µm, with a pixel size increment of 500 nm. Finally, the Iterative Fourier Transform Algorithm (IFTA) was adopted for the design and optimization of MC‐DOE. The simulations of the components working at 532 and 445 nm were performed in a similar way.

*Silicon Master Fabrication*: First, silicon oxide with 602 nm was thermally grown on a 4 in. silicon wafer. It was carried out by a dry–wet–dry thermal oxidation process at the temperature of ≈1100 °C. Subsequently, standard photolithography was performed with LC100A photoresist, and then the microstructures of the MC‐DOE were defined by the DOE mask. Then, the silicon oxide was etched with the depth of 602, 493, and 413 nm, respectively, in the three components by reactive ion etching (RIE). Finally, the photoresist was removed by a wet etching process using H_2_SO_4_.

*In vitro Glucose Detection and Drugs Release Characterization*: In Figure 3a, the Silk MC‐DOE with the relatively optimal thickness of ≈45 µm (Figure S12, Supporting Information) was separately doped with HRP, glucose, and glucose oxidase (GO*x*) in its three components (labeled as Component I, Component II, and Component III). Each component was water annealed for different time (i.e., component I, water annealing for 2 h; component II, water annealing for 4 h; component III, water annealing for 2 h) to obtain desired degradation rate. The crystallinity level of silk DOE shows no appreciable variation in its refractive index or diffraction performance (Figures S13 and S14, Supporting Information). The silk MC‐DOE was then immersed in 10 mL proteinase K solution (with a concentration of 0.3 mg mL^−1^) to mimic the degradation process. The degradation time of component I and component III was selected as 20 min to release appropriate amount of enzymes. The component II was degraded for 50 min, during which 600 µL glucose‐containing solution was taken out every 5 min for glucose detection. At different time points, the silk MC‐DOE was taken out from the solution for optical performance measurements. 1 g of ODA was added to 100 mL methanol and stirred to dissolve. 0.1 mL ODA solution was added to 12 mL phosphate buffer with pH of 6. Note that ODA‐containing solution should not be placed for long time. 2.5 mL o‐dianisidine‐containing solution, 600 µL glucose‐containing solution, and 600 µL of HRP‐containing solution were mixed and then heated in a 31 °C water bath for 3 min. 600 µL glucose oxidase solution was added to the mixed solution and heated in a 31 °C water bath for 2 min. Then, the absorbance of glucose‐containing enzymes solution was measured at 460 nm according to a precalibrated absorbance–concentration curve (Figure S9, Supporting Information). A gradient concentration (from 1 to 12 mg mL^−1^) of glucose solution was prepared and the relationship between the concentration and the corresponding OD value was plotted (Figure S10, Supporting Information). By calibrating the mixed solution obtained at different time points by the standard concentration of glucose solution, a one‐to‐one correspondence between *R* value and concentration of glucose solution at different time points could be obtained, thereby establishing a model for glucose detection.

*Quantitative Evaluation of Infection Situation*: 500 µL physiological saline was added to the back of experimental rats and then ≈50 mg of infected tissues was harvested from the back of experimental rats at the infection sites to be grounded into homogenate. Then, a series of 100 µL diluted solution (10^2^ times, 10^4^ times, and 10^6^ times, respectively) was inoculated into the culture dish and cultured for 24 h before obtaining the effective colony count of the *E. coli* and *S. aureus*.

*Animal Experiments*: The rats were purchased from SLAC Laboratory Animal (Shanghai). All the rats are male with average weight of 120 g feeding in the Department of Laboratory Animal Science for 5 d before experiment.All animal experiments were conducted in accordance with approved Institutional Animal Care and Use Committee (IACUC) protocols at Huashan Hospital of Fudan University (20110307‐020). The back skin of a rat was cut to create a wound that was subsequently infected by *S. aureus* and *E. coli*. The silk MC‐DOE was placed at the infection side with the microstructures facing down. The silk MC‐DOE was illuminated respectively by laser points with desired wavelengths with an incident angle of ≈10° for monitoring the reflective diffraction patterns. After 30 min, the silk MC‐DOE was removed and the wound was stitched.

*Transmission Experimental Setup for MC‐DOE Characterization*: The MC‐DOE was illuminated by three laser pointers (using a laser diode emitting a narrow band coherent laser beam of visible light). The distance between the MC‐DOE and projection plane was fixed to be 1.5 m and there were no collimating/focusing lens in this experiment.

## Conflict of Interest

The authors declare no conflict of interest.

## Supporting information

Supplementary
