# Supplementary material for: Programmable Vanishing Multifunctional Optics
Source: Adv Sci (Weinh). 2018 Dec 27;6(4):1801746. doi: 10.1002/advs.201801746 (PMC6382307; doi:10.1002/advs.201801746)
Supplement: Supplementary file 1 — Supplementary [file ADVS-6-1801746-s001.pdf]

Copyright WILEY-VCH Verlag GmbH & Co. KGaA, 69469 Weinheim, Germany, 2018.

ADVANCED SCIENCE
Open Access

Supporting Information

for *Adv. Sci.,* DOI: 10.1002/advs.201801746

Programmable Vanishing Multifunctional Optics

*Xiaoqing Cai, Zhitao Zhou, and Tiger H. Tao**

Copyright WILEY-VCH Verlag GmbH & Co. KGaA, 69469 Weinheim, Germany, 2013.

Supporting Information

Programmable Vanishing Multi-functional Optics

Xiaoqing Cai, Zhitao Zhou, Tiger H. Tao*

Xiaoqing Cai, Dr. Zhitao Zhou, Prof. Tiger H. Tao
State Key Laboratory of Transducer Technology, Shanghai Institute of Microsystem and Information Technology, Chinese Academy of Sciences, Shanghai, 200050, China
E-mail: tiger@mail.sim.ac.cn

Xiaoqing Cai, Prof. Tiger H. Tao
School of Graduate Study, University of Chinese Academy of Sciences, Beijing 100049, China

Prof. Tiger H. Tao
School of Physical Science and Technology, ShanghaiTech University, Shanghai 200031, China

Keywords: programmable vanishing, multi-chromatic, multi-level, diffractive optical elements

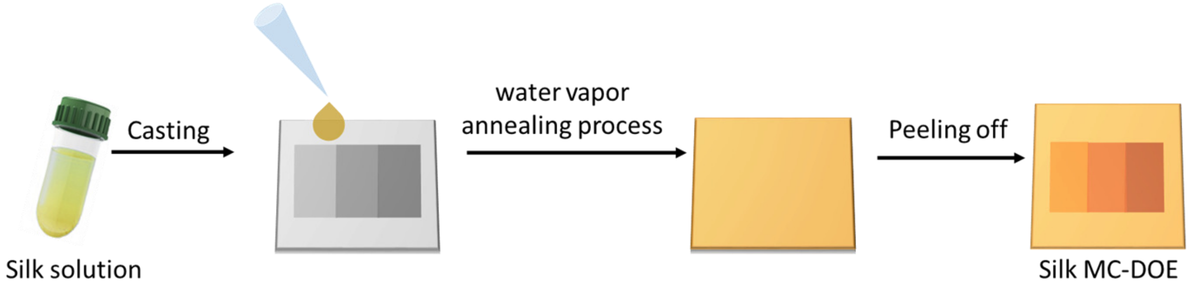

Figure S1. Fabrication process: The microstructures on the surface of silicon MC-DOE can be precisely transferred to the silk protein film using a "cast-and-peel" soft lithography technique. The crystallinity of silk components can be regulated by a water vapor annealing process.

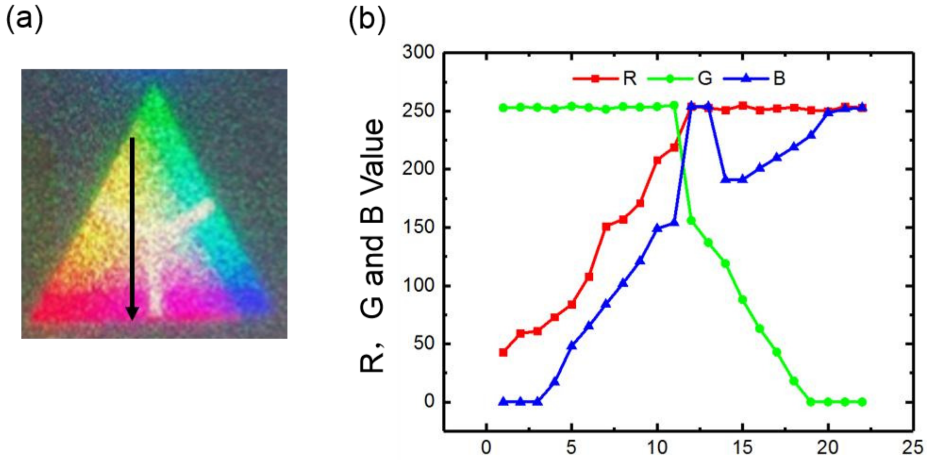

Figure S2. (a) The performance of multi-chromatic diffraction pattern is evaluated by the RGB color model along the arrow direction. (b) By illuminating the components of MC-DOE with laser points working at corresponding wavelengths (i.e., 650 nm, 532 nm and 445 nm), three curves reflecting 256 color levels have been achieved.

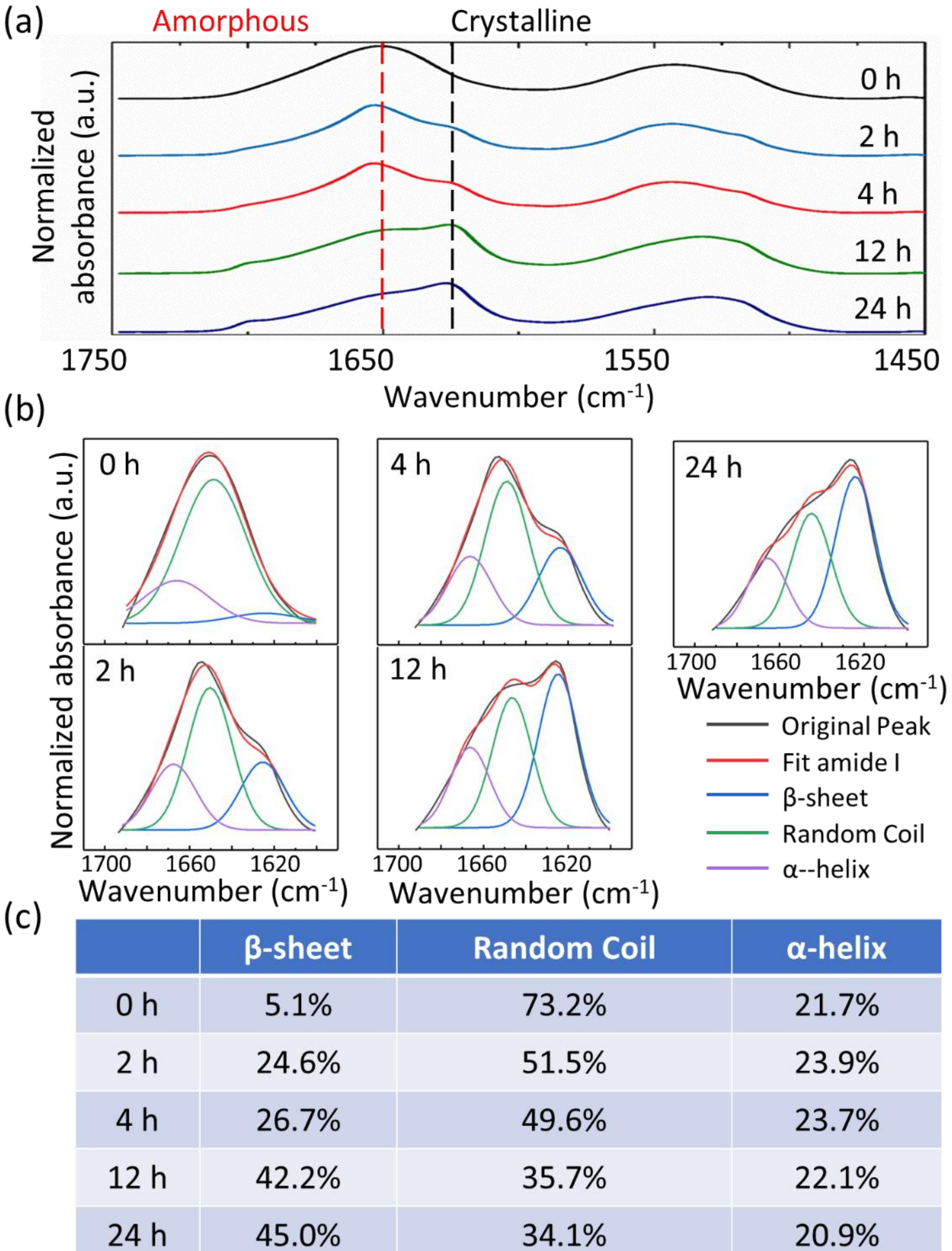

(c)

	β-sheet	Random Coil	α-helix
0 h	5.1%	73.2%	21.7%
2 h	24.6%	51.5%	23.9%
4 h	26.7%	49.6%	23.7%
12 h	42.2%	35.7%	22.1%
24 h	45.0%	34.1%	20.9%

Figure S3. (a) FTIR spectra of silk films treated with different duration of water-annealing. (b) & (c) Quantification of the FTIR spectra in (a) using deconvolution.

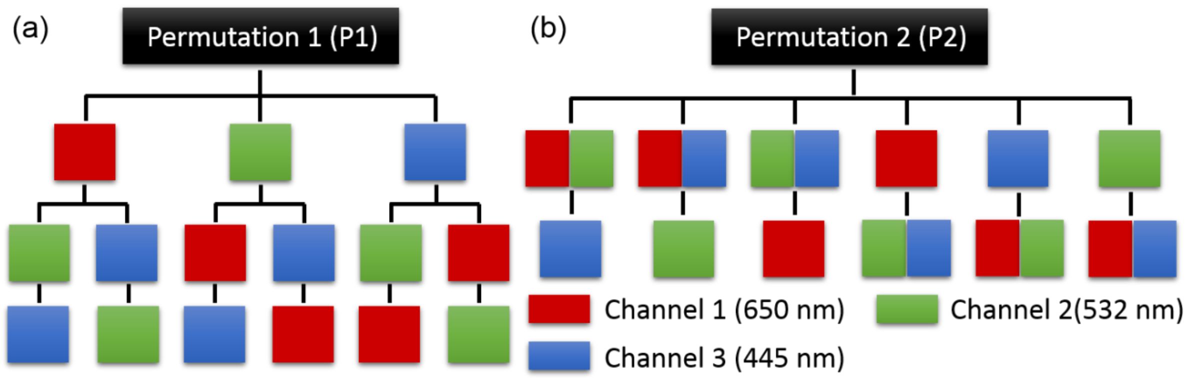

Figure S4. The information channel permutation can be classified into three types based on the number of channels that are turned on or off in a single time. (a) In permutation 1, single channel has been chosen to treat the multi-level information. (b) In permutation 2, two channels are opened or closed simultaneously. In the last permutation (It has not been shown), all channels are opened or closed at the same time.

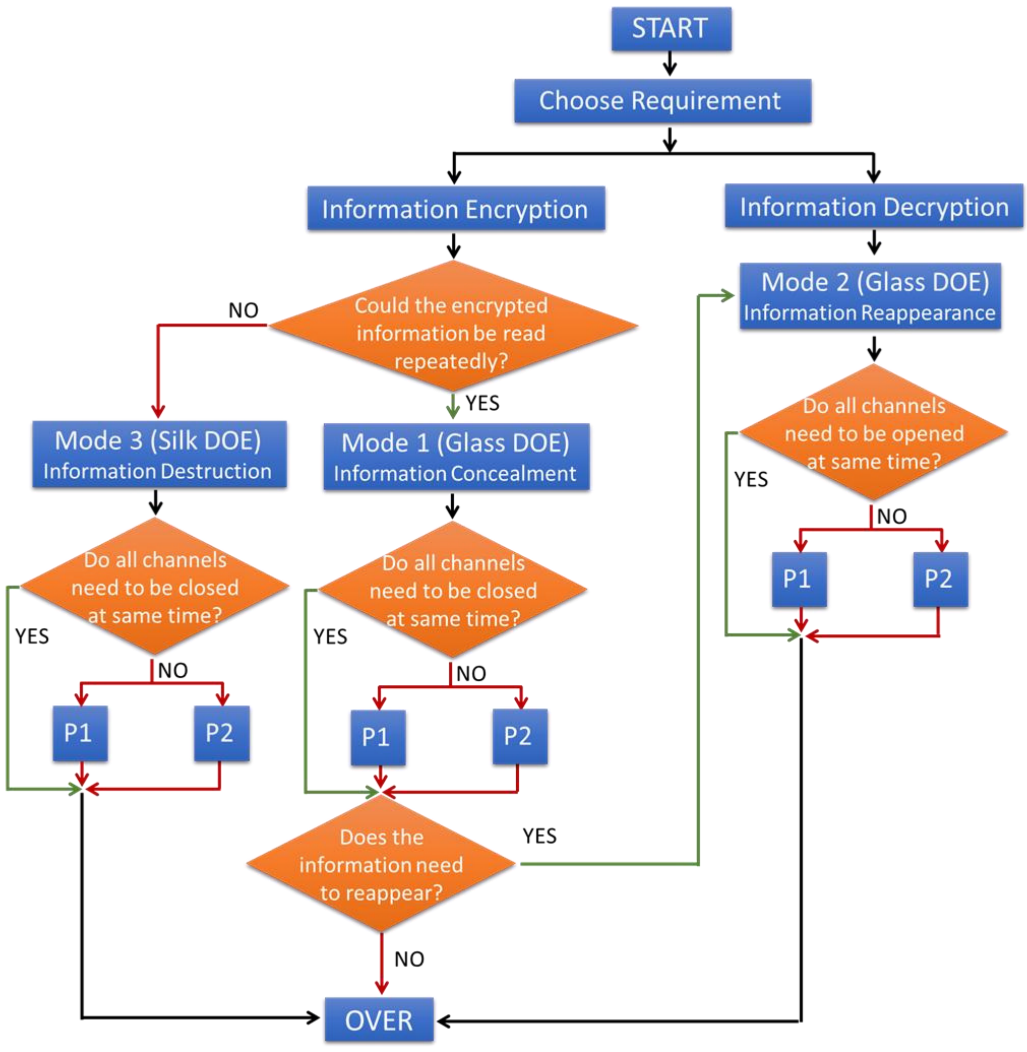

Figure S5. The flow chart is programmed according to the requirement of the information processing. Three operation modes of MC-DOE are the same as Figure 2.

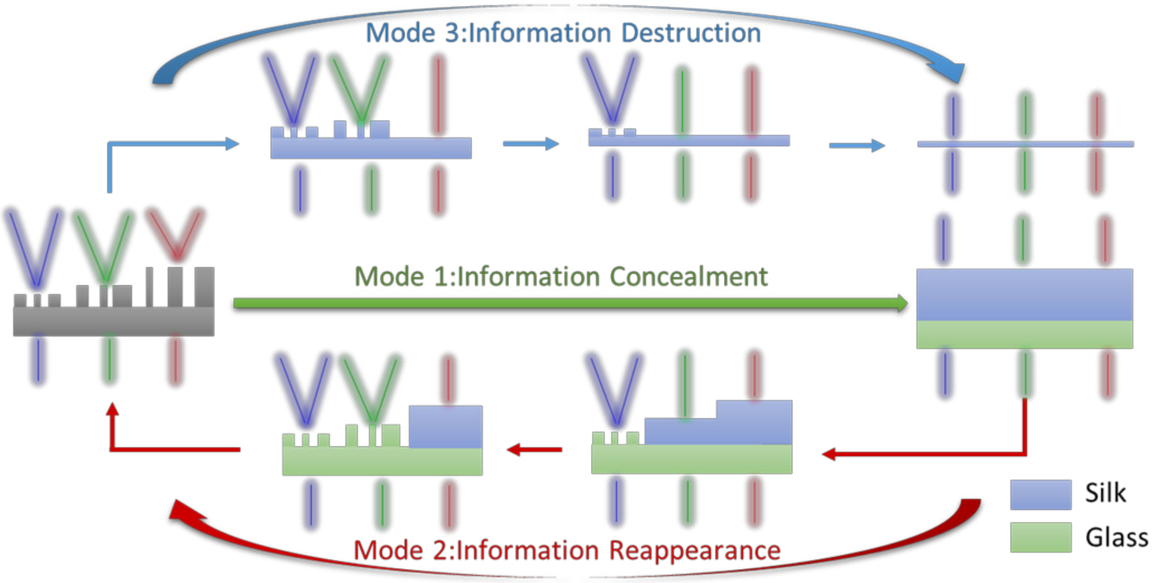

Figure S6. Schematic diagram of three work modes for information processing. Mode 1 and mode 2 are reversible by selectively coating or removing the silk layer on glass MC-DOE in order to realize information concealment and reappearance. The part or entire destruction of multi-level information (mode 3) stored in silk MC-DOE can be realized by degrading the microstructures of the components with different crystallinity.

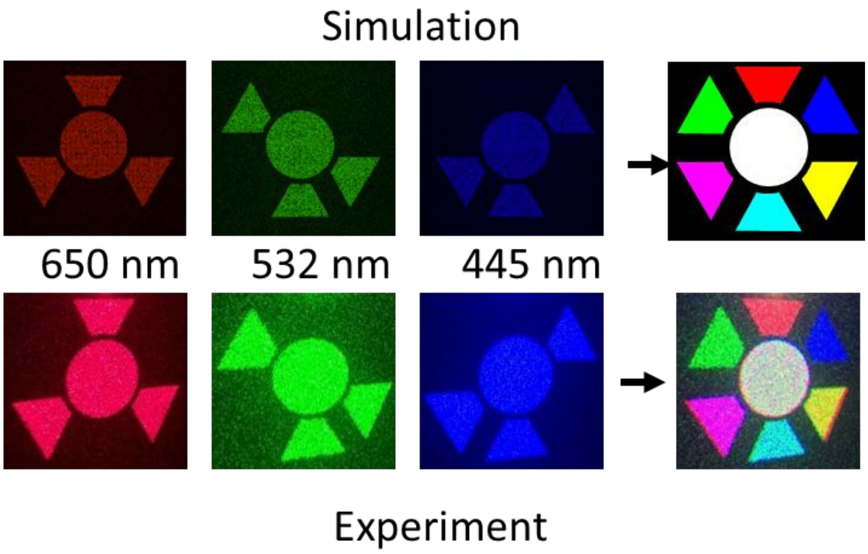

Figure S7. Simulated (Top) and experimental (Bottom) results of the silk MC-DOE illuminated by three laser pointers with different wavelengths (650 nm, 532 nm and 445 nm).

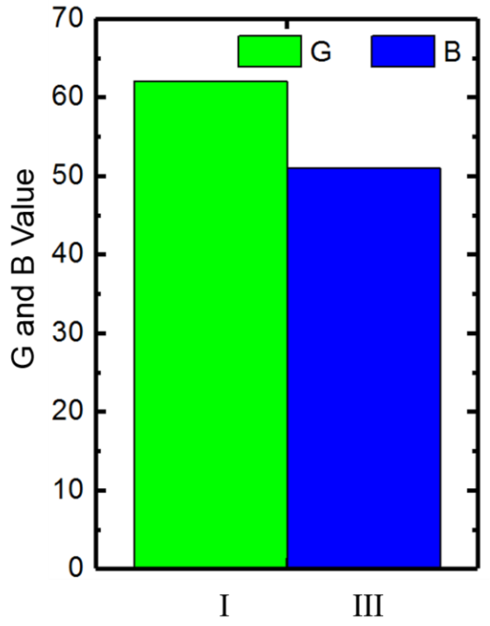

Figure S8. Monitoring G value of component I and B value of component III to get the appropriate amount of enzymes.

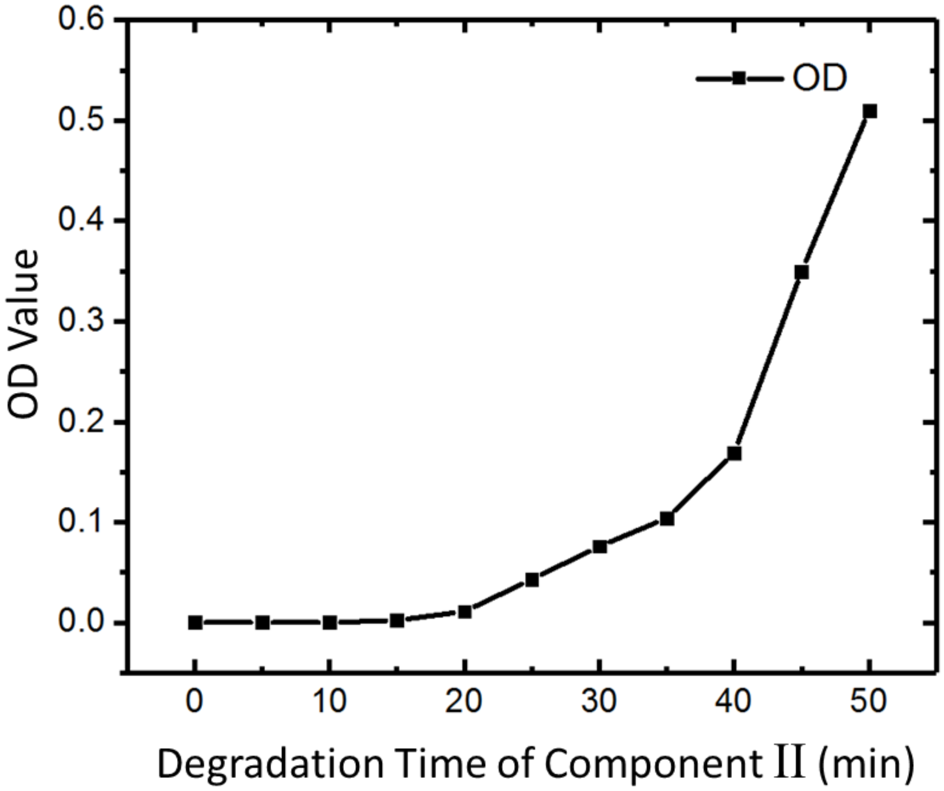

Figure S9. As component II gradually degrades, the OD value of the glucose-containing solution increases.

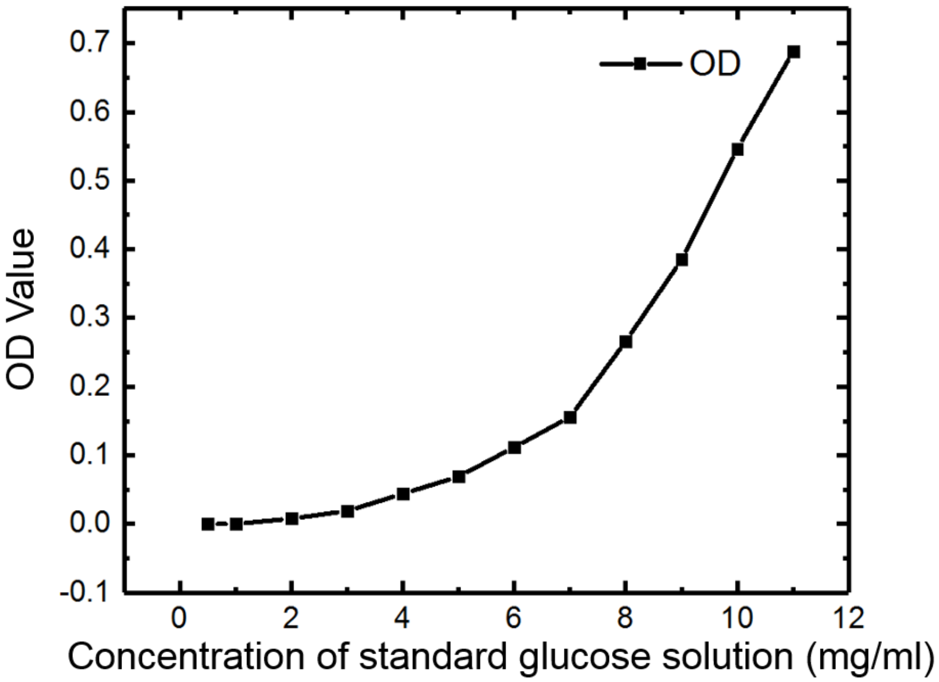

Figure S10. As the concentration of glucose solution gradually increases, the OD value of the glucose-containing solution increases.

	Component I	Component II
Group 1	none	None
Group 2	Gentamicin	none
Group 3	none	Metronidazole
Group 4	Gentamicin	Metronidazole

Figure S11. Four sets of controlled trials were designed to explore the therapeutic effect of multiple antibiotics with four groups on complex infections caused by multiple pathogens.

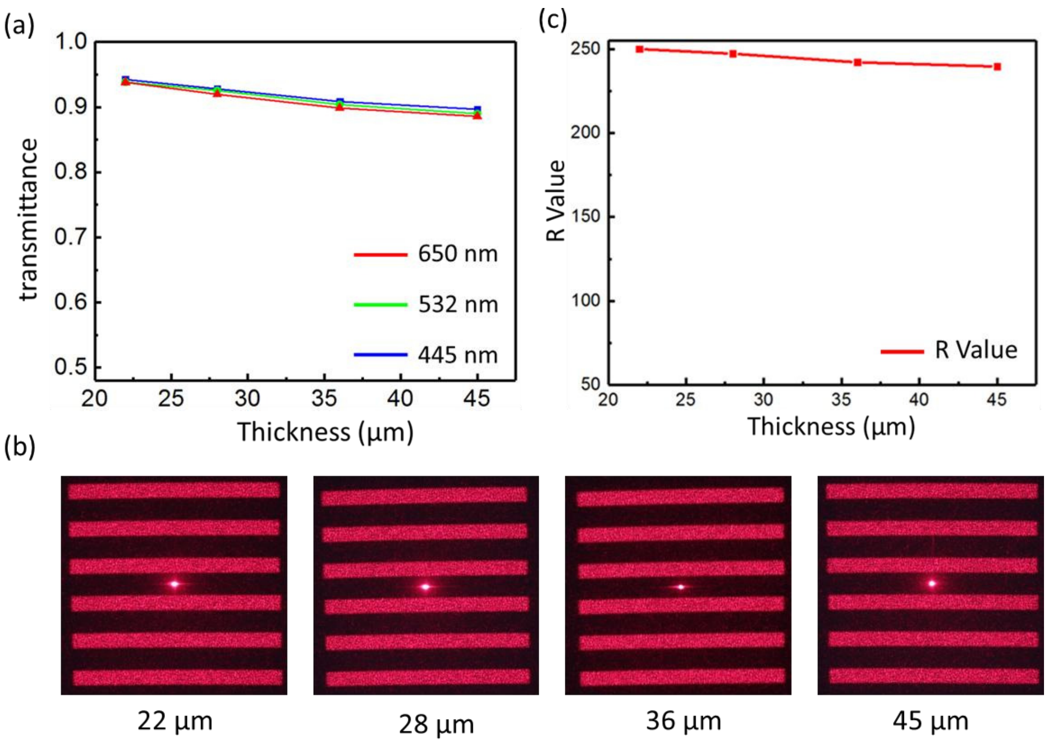

Figure S12. (a) The light transmittance of the flat silk films with different thickness. The (b) diffraction patterns and (c) corresponding R value of silk MC-DOEs with different thickness at the working wavelength of 650 nm. The thicker thickness of silk MC-DOEs results in lower transmittance (i.e., worse diffraction performance). However, ultrathin silk film cannot be manipulated effectively for processing. Experiment results indicate ~45 μm is a relatively optimal thickness, which shows satisfactory diffraction performance and can be manipulated effectively.

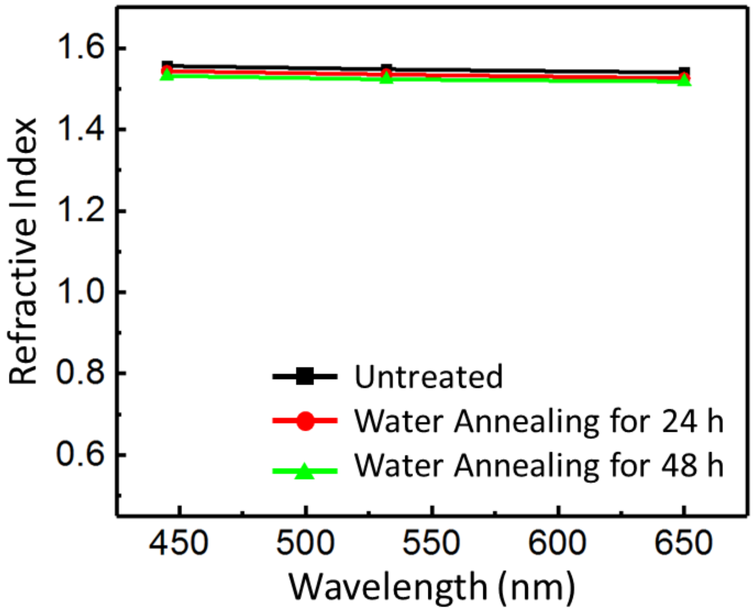

Figure S13. Refractive indices of identical silk films with different degree of crystallinity.

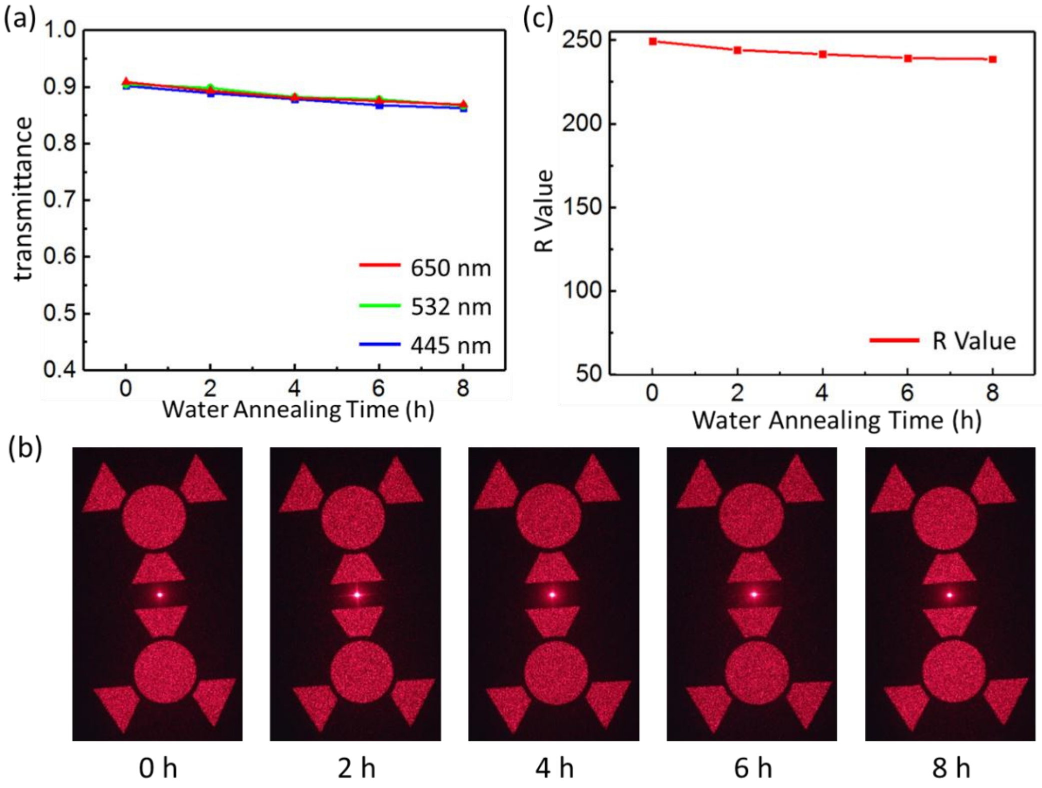

Figure S14. (a) The light transmittance of the flat silk films with different crystallinity levels. The (b) diffraction patterns and (c) corresponding R value of silk MC-DOEs with different crystallinity levels at the working wavelength of 650 nm.
